# Life on the frontlines: Treating COVID-19 patients as an internal medicine resident physician in New York City

**Published:** 2020-08-01

**Authors:** Gurchetan S. Randhawa

**Affiliations:** Department of Medicine, Maimonides Medical Center, Brooklyn, New York, USA

**Keywords:** ARDS, COVID-19, internal medicine, resident physician

Many have asked what the situation is like as a physician trying to combat the coronavirus disease 2019 (COVID-19) on the frontlines in one of the most devastated areas in the world. What I can tell you is that this has been one of the longest, most exhausting chapters in my life. I was one of the first physicians to treat a patient infected with COVID-19 at Maimonides Medical Center in Brooklyn, New York, in March of 2020, and continue to treat COVID-19 patients on a daily basis. While the initial surge in New York City has ended, the residual psychological, physical, and spiritual scars remain. The virus infected many of my colleagues and some have perished as a result. Many patients have died. Despite this harrowing experience, I also witnessed an extraordinary testament to the human spirit, as the world united to fight this singular, invisible, common foe. I hope this editorial will help those fighting the pandemic – domestically in the wake of a resurgence of COVID-19 – and elsewhere across the globe.

When I first heard about COVID-19 in December of 2019, I was alarmed, but I felt like it would be something that would likely pass with time. Like *Zika* and *Ebola* before it, there would be fear of it being transmitted into the United States, but ultimately it would not amount to much. Then, the number of cases started to dramatically increase and soon the disease started to spread to neighboring countries such as Japan and South Korea, before making its way into Europe and finally, into North America. I started to think that COVID-19 would be something different and that it would inevitably reach New York City. I felt some cautious optimism that the United States had a head start to prepare and that the government was hard at work for what was coming our way. What I can tell you with confidence is that we were not prepared and not enough was done to contain the spread of the virus in its infancy in the United States.

As the pandemic approached, I started to notice small cracks in society that became worse by the day. People began to panic, as they created shortages nationwide on essential items of daily living. I remember one day I was about to get on the train and go to the hospital when I noticed something unusual: A subway car that had a section that was empty when it would be normally packed to the brim with rush hour commuters. When I boarded the train, I looked to my left and noticed the only empty seats were near Asian passengers. I was happy to sit near them without hesitation and remember getting some stares from fellow passengers. This subtle xenophobia was more pronounced with the disgusting video of the man on the MTA spraying an Asian man with a cleaning solution, while berating and yelling at him to move. Asian men and women started to get attacked and blamed for the virus – politicians argued over what to do for the looming crisis – experts disagreed on how to mitigate its spread – scientists tried to find answers that they simply did not have. Instead of coming together, COVID-19 was exposing all the fear and division in this country, which would make fighting the virus even harder: The United States was doomed from the start.

When the first cases started to arrive in New York City, we had to contact the Department of Health (DOH) and justify each test for a suspected patient. At times, physicians were at odds with the DOH and requests for testing were denied, despite physicians being able to directly see the patient and use their clinical acumen to justify a test. The numbers of testing kits themselves were scarce in number and initial testing was only available for people with a recent travel history to Hubei Province, China, despite the continuing worldwide spread. The test results would take approximately 4 days before they would come back, which also compounded the initial problems. A lack of testing kits, strict criteria for testing, in conjunction with long turnaround times for laboratory results was a recipe for disaster. What made matters worse, was that the government does not enforce quarantine if a person is exposed to someone already infected by COVID-19. I feel that many people do not realize that they should be quarantined and have been roaming outside their homes freely, spreading the disease to others, including valuable health care workers.

I remember reading about promising research studies in preparation for the inevitable COVID-19 patient that I would encounter. Little did I know that in one week I would be starting those experimental medications on actual patients. As the situation became progressively more chaotic, panic started to spread. Major announcements and dramatic changes to hospital practices occurred by the hour. Instead of the government ensuring adequate supplies of personal protective equipment (PPE), health care workers such as myself faced severe shortages of essential masks, which we need to protect ourselves against patients infected with COVID-19. I felt like we were abandoned and left to fend for ourselves. It was unfathomable to think that some health care workers in the United States were reduced to wearing trash bags for PPE at the beginning of the pandemic, as documented by the press. With a lack of PPE, every single health care worker fears getting exposed and dying. During the initial surge in March, I had to use the same N95 mask for several weeks due to severe shortages and developed an irritant skin reaction on the bridge of my nose. I subsequently developed a blister that was excruciatingly painful. I would come home every night and wash the blood and purulent discharge off my face. I now have a permanent scar that was left behind and it serves as a daily reminder of that dark chapter in my life. As the crisis worsened, more and more patients started to arrive.

I started to see people that were going about their daily lives suddenly become progressively more short of breath and hypoxic, to the point of intubation in a matter of days. Just like AIDS in the 1980s, we started to notice a pattern in chest imaging: Instead of seeing *Pneumocystis jiroveci* pneumonia in AIDS patients, we started to see a pattern of rapidly progressing acute respiratory distress syndrome (ARDS) with ground-glass findings on chest computerized tomography (CT) imaging. In addition to ARDS and chest CT imaging, we noticed patients often had elevated inflammatory markers, as well as lymphopenia, with routine laboratory work. Painful discussions were made with families regarding treatments, prognoses, and eligibility to join promising trials such as Gilead’s initial remdesivir trial. Unfortunately, not all patients were eligible to join the experimental trial due to multiorgan failure and a poor prognosis. You can imagine the pain the family members felt when hearing the news that their loved one could not join the clinical trial for a medication that the world hoped would be an effective therapy for COVID-19. We feared the same situation that occurred in Italy, where physicians had to make the impossible decision of deciding who gets a ventilator and who does not, ensuring that one patient will inevitably die, due to the shortage of hospital beds. Without any official treatment guidelines, physicians relied on initial published scientific literature and used their own clinical judgment to see which therapy they would like to start. We were certainly in uncharted waters.

COVID-19 was much more than a disease, as it had tremendous socioeconomic ramifications. We started to see patients who lost their jobs and resorted to alcohol as an escape from their economic woes. Many were treated for alcohol withdrawal and intoxication during this time. I also felt that the virus disproportionately affected the indigent. I recall one patient encounter that stayed with me for a very long time. I treated an elderly Chinese woman who was bedbound, but was exposed to the virus through a family member who unknowingly contracted the virus. After calling and notifying the family of the results and advising quarantine precautions, I also called the patient’s home health aide (HHA), who was in close contact with the patient. As I informed the HHA of the results, she suddenly started to cry and was inconsolable. Through a Cantonese interpreter, I asked her what was wrong: “Doctor, I was a nurse in Hong Kong during severe acute respiratory syndrome in 2003, and I know what this virus can do. I know that I will die, but I do not want to expose my family. My husband is recovering from surgery and my parents are in poor health. We are poor and live in a washroom all living close together. There is no way that I can isolate myself after being exposed while also caring for my family. I tried calling 3-1-1 to receive testing, but I cannot find a test for myself. Doctor, what should I do?” I had to pause, collect myself, and struggled to maintain my composure. I never felt so powerless in my life. There is no prescription for poverty.

One of the most difficult moments was when there were multiple cardiac arrest codes called at the same time. As I entered a patient’s room to perform cardiopulmonary resuscitation (CPR), another overhead announcement would come for another patient in cardiac arrest. Eventually, there were five cardiac arrest codes that were called at the same time. I felt overwhelmed and had to decide which patient had the most likely chance of survival, since I could only perform CPR on one patient at a time. We were able to revive one patient and we left the room feeling ecstatic. That happiness was short-lived as that patient, along with the other four patients in cardiac arrest, all eventually died. As I performed CPR, I thought about all of the viral particles surrounding my colleagues and myself as the patients struggled to breathe. I was always fearful of being infected by the virus in this high-risk environment. This trauma was repeated dozens of times every day as the 1^st^ year resident physicians performed CPR on COVID-19 patients. We dreaded hearing the overhead page to respond to cardiac arrest codes, as the outcome was always the same: Death.

I remember going into the emergency department (ED) to admit new patients into the medicine service and was horrified with what I saw. The ED was at maximum capacity and all around me were patients struggling to breathe. Patients rushed into the ED on stretchers in respiratory distress. CPR was performed on patients in the resuscitation rooms as they died one by one. The look of despair of the patients in conjunction with the deafening sounds of the alarms put me into a shock. There is a certain look that COVID-19 patients have when they are struggling to breathe – a look of exhaustion, defeat, and impending death. Eventually, there were so many deaths that the hospital acquired several refrigerated trucks to store the deceased outside. They served as a grim reminder of the dangers that we faced inside. Every day I woke up afraid of coming down with a fever. I knew how it would end. Will I ever see my family again? Or will I die on a hospital stretcher, alone, in the middle of the night, surrounded by the weary, exasperated health care workers who tried their best to revive me. Will I be placed in a body bag and be put in one of the refrigerated trucks outside? It dawned on me that this nightmare was a sobering reality suffered by many at our hospital.

During one of my shifts in the medical intensive care unit (MICU) at the beginning of the crisis, we had a patient that needed to be tested for COVID-19 because he had a roommate at home that had flu-like symptoms. Either one of the senior physicians or myself had to go inside the room to test him. The senior MICU physician volunteered to go inside the room and test the patient as I waited outside. Afterward, I spoke to him, expressing how I look up to him, and that I felt like I let him down. I told him that it should have been me that went inside the room, not him, because he has a wife and young children, and that the hospital needs him more than they need me due to his experience. The senior pulled me aside, shook my hand firmly, grabbed my shoulder, and told me that he appreciates it, that I am a great resident, and I am helping the team by continuing to fight. I will never forget this moment for the rest of my life.

I saw him and the other seniors walk into rooms without hesitation to place invasive lines and to do whatever they could do for the patients given the dire circumstances. It was the courage and sacrifice I saw on a daily basis, not just from my fellow physicians, but from the entire hospital staff, that kept me going.

As the crisis progressed, New York City became the epicenter of the pandemic. Our institution was forced to rapidly expand and create new, innovative ways to combat the growing pandemic:

1. **Space:** New intensive care unit units were created and a hospital that was formerly closed was reopened to care for COVID-19 patients with a lower acuity of care. It is important to be creative and to think of new ways to increase the amount of space a hospital can provide additional beds during a surge. We also used our cancer center as well as an adjacent rehabilitation center to create additional space for new hospital beds.
2. **Flexibility:** Eventually, other departments such as emergency medicine, general surgery, psychiatry, orthopedic surgery, pediatrics, Ob/GYN, primary care, and the internal medicine subspecialties, all worked with the Department of Medicine, to unite together and fight COVID-19. It was a very special moment in the history of our institution, to see residents from the Department of Medicine leading all of the different multidisciplinary teams against a common enemy. As a member of the Department of Medicine, I felt incredibly proud of the work that my colleagues accomplished during this time. This flexibility and cross-discipline cooperation were paramount to our initial success at combating the virus.
3. **Communication:** The initial surge of a pandemic can be chaotic and effective communication is necessary. Important changes to hospital policy must be effectively disseminated and easily understood by staff. Town hall meetings, morning huddles, E-mail, and an easily accessible central electronic COVID-19 hospital resource center were some of the techniques that enabled effective communication.
4. **Exposure:** Decreasing staff exposure is vital, as health care workers are an extremely valuable resource. To combat fatigue as well as mitigating staff exposure, we implemented a pandemic staff schedule, utilizing smaller teams with longer shifts. Internal medicine resident physicians worked either 3 or 4, 12 h shifts a week, with either 3 or 4 days off. This additional time off enabled our staff to be better rested and decreased exposure. We placed oxygen monitors and other equipment outside of rooms so that health care workers can access medical equipment without having to enter COVID-19 rooms to further prevent staff exposure. We also utilized video conferencing to hold town hall meetings and educational events in lieu of face-to-face meetings. Visitation hours were suspended except in emergency situations, to also decrease exposure to staff, as well as our patients. We developed a call center, whereby family members can receive updates on the status of their loved ones. Patients with confirmed or suspected COVID-19 were isolated from patients that were confirmed to be negative. Staff members were required to wear PPE at all times to further prevent the transmission of the virus. By decreasing exposure, it increases patient safety and ensures that staff members are healthy and able to contribute to the fight against COVID-19.
5. **Health and well-being:** The health and well-being of the staff is also critical during a pandemic. We had confidential psychiatric counseling and mental health services available for all members of the hospital. We also had assistance for transportation and housing. We had visiting healthcare workers from across the United States travel to New York City to provide additional support for our staff. Testing for COVID-19 for employees was limited at the start, however with time, it was more readily available. We received cheers at 7 PM from fellow New Yorkers as we left our shifts and returned home to rest. We received generous donations from local businesses and organizations, but also from all over the world, ranging from medical supplies, to food, and other provisions. Hazard pay was extended to health care workers. Famous celebrities sent thank you videos to our staff. The exploits of the staff were documented in the press and with the media. All of these gestures, small and large, boosted staff morale, which is vital to maintain in the wake of a pandemic.

While these measures were important to our hospital’s success at combating the virus, the unfortunate reality of the pandemic’s effect on society applied outside of the hospital. My younger brother used to drive 2–3 h from my family’s home in Connecticut, just to bring food and supplies for me, when I could not obtain them locally. Some of the most poignant moments were when he would drop off the box of goods at my apartment building’s front door, but I could only say good bye to him through the glass in-between us. Since I was a physician with a high chance of exposure, I could not risk any transmission to my family. I felt like I was living in an alternative world devoid of human contact and connections, which are essential to the human condition. Eventually, as the crisis started to subside, I received a negative COVID-19 test and was able to go home to my family for the 1^st^ time in 6 months. Instead of hiding behind the glass, I gave my younger brother a heartfelt embrace and shared this emotional moment that we will remember for the rest of our days. My family was proud of me and discussed how our small hometown held a large motorcade in front of our home as a gesture of gratitude for my efforts in combating COVID-19. I felt a tremendous amount of gratitude for this gesture. As I rested in the comforts of my home, I reflected on all that I endured during the pandemic. Could I have done more? Where do I draw the line between service and self-preservation, the so-called “moral injury?” I thought about all of the lives that were taken but also the lives that were spared. I grew angry with the people who refuse to believe that the virus exists and refuse to comply with mask wearing. After risking my life and witnessing the devastation caused by the virus, it was an insult to all that we sacrificed during this time. I thought about the grave injustice that was done to American health care workers, as we sent our brave sons and daughters into a war without the proper supplies. How many of their lives could have been spared with the proper equipment? I thought about the health care workers around the world who died and never got a chance to return back home like I did. I thought about the special bond – the camaraderie – that I formed with my colleagues that will link us together for the rest of our lives.

As I sat outside on my deck with my family, I looked at the sunset on this beautiful summer night and saw dozens of fireflies. As the darkness of the night approached, the fireflies remained, illuminating the night sky for all of us. In a world that is full of darkness, it is important to remember the moments of light, with each firefly representing a united humanity and the souls of those that perished from this disease. May their legacies live on forever and their light guides us in these uncertain times.

For my fellow health care workers as well as scientists around the globe, the eyes of the world are upon you. It is up to us to figure out how to defeat this pandemic and prevent further death and suffering. We are limited in our supplies but not in our courage and ability to support each other. The fight is only beginning, but one day we will emerge victorious.

## Dedication

The biggest loss for our hospital was the passing of our beloved leader, Dr. Stephan Kamholz, M.D., due to complications suffered from COVID-19. This editorial is dedicated in his honor. Dr. Kamholz was the Chairman of the Department of Medicine and one of the most accomplished and brilliant educators that I have ever known. He was also exceptionally kind and humble, despite all of his accolades. Even during the crisis, he never stopped teaching, and always cared for the well-being of his staff. He had a sharp wit and a great sense of humor that will be missed. His steadfast leadership and courageous actions during the COVID-19 crisis saved countless lives. He will always be remembered as a model physician and a hero in my eyes. We will always be in debt to his service and sacrifice in the worst crisis our hospital has ever known. May he rest in peace and may his memory inspire us to be courageous and selfless as we continue to fight the battle against COVID-19.

Stephan Kamholz, M.D.

Gurchetan S. Randhawa, MD

